# The Relationship Between Big Five Personality and Social Well-Being of Chinese Residents: The Mediating Effect of Social Support

**DOI:** 10.3389/fpsyg.2020.613659

**Published:** 2021-03-05

**Authors:** Yanghang Yu, Yuanyuan Zhao, Dongyan Li, Jingqiu Zhang, Jiewei Li

**Affiliations:** ^1^School of Public Finance and Management, Yunnan University of Finance and Economics, Kunming, China; ^2^Tourism and Cultural Industry Research Institute, Yunnan University of Finance and Economics, Kunming, China; ^3^International College, National Institute of Development Administration, Bangkok, Thailand; ^4^National Centre for Borderlands Ethnic Studies in Southwest China at Yunnan University (NaCBES), Yunnan University, Kunming, China

**Keywords:** big five personality, social support, social well-being, China, mediating effect

## Abstract

Previous studies have noted that personality traits are important predictors of well-being, but how big five personality influences social well-being is still unknown. This study aims to examine the link between big five personality and five dimensions of social well-being in the Chinese cultural context and whether social support can play the mediating effect in the process. This study included 1,658 participants from different communities in China, and regression analyses were conducted. Results revealed that five personality traits were significantly related to overall social well-being; extraversion was significantly related to social integration; agreeableness was positively related to all five dimensions of social well-being; conscientiousness was positively related to social actualization, social coherence, and social contribution; neuroticism was negatively related to social integration, social acceptance, social actualization, and social coherence; openness was positively related to social integration, social acceptance, social coherence, and social contribution. Social support plays mediating roles in the relationships between extraversion/agreeableness/conscientiousness/neuroticism/openness and social well-being, respectively.

## Introduction

Personality variables are strong predictors of well-being, a large body of research has explored the associations between big five personality and subjective well-being (DeNeve and Cooper, 1998; Gutiérrez et al., 2005). Unfortunately, the psychological construct of well-being portrays adult well-being as a primarily private phenomenon largely neglecting individuals’ social lives (Keyes, 2002; Hill et al., 2012). Individuals are embedded in social structures and communities; as such, it is necessary to evaluate one’s circumstance and functioning in a society; more attention needs to be devoted on the topic of social well-being (Keyes, 1998). Previous studies focused on the social well-being from the perspective of interpersonal factors, such as sense of community (Sohi et al., 2017), and civic engagement (Albanesi et al., 2010). However, less work has examined social well-being from the level of the individual (Keyes and Shapiro, 2004).

Although there are few studies focusing on the relationship between five personality traits and social well-being (Hill et al., 2012; Joshanloo et al., 2012), their data come from United States or Iran; Chinese cultural background has been conducted to a lesser extent. Different countries have different cultural traditions. Personality is created through the process of enculturation (Hofstede and McCrae, 2004). The interplay of personality and cultural factors was found to predict residents’ well-being significantly (Diener and Diener, 1995). Confucius culture has embedded itself in the daily life of the Chinese, however, studies about the relationship between personality and social well-being under the context of Chinese culture are largely overlooked.

In addition, present studies (Hill et al., 2012; Joshanloo et al., 2012) examine only the direct effect of personality on social well-being. The mechanism between big five personality and five dimensions of social well-being has been neglected. Additionally, social support can help individuals protect against the health consequences of life stress and increase their well-being (Cobb, 1976; Siedlecki et al., 2014). Thus, following a social support perspective, the present study examined not only the relationship between five personality traits and domains of social well-being, but also whether social support can play a mediating effect in the relationship between big five personality and social well-being.

## Literature Review and Hypothesis

### Big Five Personality and Social Well-Being

The big five personality consists of five general traits: extraversion, neuroticism, openness, agreeableness, and conscientiousness (John and Srivastava, 1999). Extraversion refers to the degree to which one is energetic, social, talkative, and gregarious. Agreeableness reflects the extent to which one is warm, caring, supportive, and cooperative and gets along well with others. Conscientiousness involves the extent to which one is well-organized, responsible, punctual, achievement-oriented, and dependable. Neuroticism means the degree to which one is worry, anxious, impulsive, and insecure. Openness reflects the degree to which one is imaginative, creative, curious, and broad-minded (Barrick et al., 2001; Funder and Fast, 2010). Many scholars assessed personality under different culture context by a combined emic–etic approach (John and Srivastava, 1999; Cheung et al., 2001). Even if there were researches that demonstrated several unique dimensions of personality under the Chinese culture (Cheung et al., 2001; Cheung, 2004), the generalizability of the big five trait taxonomy in China is still confirmed (Li and Chen, 2015; Minkov et al., 2019). Previous studies have consistently demonstrated that the big five are associated with subjective well-being (DeNeve and Cooper, 1998; Gutiérrez et al., 2005), however, the findings are mixed under different cultural context. For instance, Ha and Kim (2013) found openness has a positive effect on subjective well-being in South Korea residents, whereas another study by Hayes and Joseph (2003) in England found that openness was not associated with each of the three measures of subjective well-being.

Culture variables can explain differences in mean levels of well-being (Diener et al., 2003). With the uniqueness of Confucian cultural tradition and social setting, it is noteworthy to discuss the relationship between personality and well-being in Chinese cultural background, especially social-well-being.

Individuals are embedded in social structures. They need to face social challenges and evaluate their life quality and personal functioning by comparison to social criteria (Keyes and Shapiro, 2004). However, the research about social well-being has been almost completely neglected in the hedonic and psychological well-being models (Keyes, 2002; Joshanloo et al., 2012). Keyes (1998) proposed social well-being, which indicates to what degree individuals are functioning well in the social world they are embedded in. Social well-being can be described on multiple dimensions, including social integration, social contribution, social acceptance, social coherence, and social actualization. Social integration is the extent to which people feel commonality and connectedness to their neighborhood, community, and society. Social contribution refers to a value evaluation that one can provide to the society. Social acceptance entails a positive view of human nature and believes that people are kind. Social coherence refers to the perception of the quality and operation of the social world and reflects a belief that society is meaningful. Social actualization is the evolution of the potential and of society and includes a sense that social potentials can be realized through its institutions and citizens. In summary, social well-being emphasizes individuals’ perceptions of and attitudes toward the whole society. Prior studies have found the effect of sense of community (Sohi et al., 2017), and social participation (Albanesi et al., 2010) on social well-being, Also, some studies have shown the outcomes of social well-being, such as anxiety problems (Keyes, 2005), general mental and physical health (Zhang et al., 2011), and prosocial behaviors (Keyes and Ryff, 1998). Personality traits and cultural factors are important predictors of well-being (Diener et al., 2003). However, the only studies about personality and social well-being were conducted in Iran or United States. It is still not known whether the association would be similar in a different cultural context (Hill et al., 2012; Joshanloo et al., 2012). For example, with the data from the MIDUS sample, Hill et al. (2012) found social well-being is positively related to extraversion, agreeableness, conscientiousness, emotional stability, and openness. In addition, previous studies did not test the correlation between five personality traits and five domains of social well-being entirely (Joshanloo et al., 2012). Personality shapes many of the attitudes and behaviors that form Keyes’ different dimensions of social well-being. Thus, certain personalities would predict social well-being; for example, extraverted persons should be more socially integrated, whereas agreeable individuals should possess higher levels of social acceptance. Based on the above, we hypothesize the following:

Hypothesis 1_a_: Extraversion is positively related to social well-being.
Hypothesis 1_b_: Agreeableness is positively related to social well-being.
Hypothesis 1_c_: Conscientiousness is positively related to social well-being.
Hypothesis 1_d_: Neuroticism is negatively related to social well-being.
Hypothesis 1_e_: Openness is positively related to social well-being.

### The Mediating Effect of Social Support

Social support refers to individuals’ psychological or material resources from their own social networks that can assist them to cope with stressful challenges in daily lives (Cohen, 2004). It comes from a variety of sources, such as friends, family, and significant others (Taylor, 2011). Social support comprised both received and perceived social support (Oh et al., 2014; Hartley and Coffee, 2019). However, many studies showed that perceived social support is more effective at predicting residents’ mental health than the received social support (Cohen and Syme, 1985). Perceived social support indicates recipients’ perceptions concerning the general availability of support (Sarason et al., 1990), which fosters a sense of social connectedness in a network and provides resources with which to overcome obstacles in their lives (Lee et al., 2001; Chen, 2013). Social support theory emphasizes that social support is an important resource that can help individuals protect against life stress and increase their quality of lives (Cobb, 1976; Cohen and Wills, 1985). Numerous studies have explored the associations between social support and well-being, including subjective well-being (Brannan et al., 2013; Siedlecki et al., 2014) and psychological well-being (Jasinskaja-Lahti et al., 2006; Wong et al., 2007). Although Inoue et al. (2015) found social support mediated the effect of team identification on community coherence, little research has addressed the effect of social support on social well-being. The benefits of social support come into play when individuals have to deal with social challenges and problems. Individuals with high level of social supports will better face social tasks (Cox, 2000). Harmonious social relationships can help residents to satisfy their social needs, better understand, and be confident of the social world. Therefore, their social well-being will increase.

Personality traits are stable predictors of social support (Swickert et al., 2010; Udayar et al., 2018; Barańczuk, 2019). Big five personality traits are found to be related to social support. Individuals with high levels of neuroticism report greater vulnerability to stress and negative affectivity, which could decrease the availability of social support (Ayub, 2015). Individuals who score high on extraversion always seek social interactions and tend to be cheerful and friendly. The positive emotions could increase their social support (Swickert et al., 2010). Individuals with high openness to experience are characterized by greater openness to emotions, appreciation of art and beauty, intellect, and liberalism. These characteristics would be significantly related to social support (Barańczuk, 2019). Agreeableness characteristics, such as modesty, compliance, and trust, may facilitate individuals building a more extensive social support network (Barańczuk, 2019). Conscientiousness are characterized by achievement-striving, self-discipline, orderliness, and dutifulness. These tendencies can help individuals better cope with life stress, so it is positively related to social support (Ayub, 2015). Culture is an important moderator between big five personality traits and social support association, but it has been largely overlooked in previous studies (Barańczuk, 2019). Therefore, studies about the relationship between five personality traits and social support under Chinese background are needed.

Previous studies discuss only the direct effect of personality on social well-being, but it remains unknown what mechanism(s) may explain this relation. Social support plays an important stress-buffering role when individuals are under high levels of life stress (Cohen, 2004). Individuals with different levels of personality traits (extraversion, agreeableness, conscientiousness, neuroticism, openness) will form different types of social support network. Further, social support will help individuals cope with social challenges and increase their social well-being. Based on the above, we hypothesize the following:

Hypothesis 2_a_: Social support mediates the relationship between extraversion and social well-being.
Hypothesis 2_b_: Social support mediates the relationship between agreeableness and social well-being.
Hypothesis 2_c_: Social support mediates the relationship between conscientiousness and social well-being.
Hypothesis 2_d_: Social support mediates the relationship between neuroticism and social well-being.
Hypothesis 2_e_: Social support mediates the relationship between openness and social well-being.

## Materials and Methods

### Participants and Procedure

Community residents from five different districts in Kunming, Yunnan Province, were selected as participants by stratified random sampling technique. Four hundred questionnaires were distributed to each district. Participants would complete the questionnaires in a face-to-face interaction with an enumerator who helped them to answer the questionnaire that was in paper format. When we administered the survey, we emphasized that the data were collected for research purposes. Participants were encouraged to answer all the questions honestly and were reminded that their responses would be anonymous. Upon completion of answering the questionnaire, participants received a small gift (e.g., tissue) as compensation for their participation. A total of 2,000 questionnaires were distributed, and 1,721 responded. After dropping incomplete and invalid data, 1,658 respondents remained. The final sample consisted of 932 females (56.2%) and 726 males (43.8%), aged 18–81 years (mean = 30.73 years, SD = 11.98 years).

### Measures

#### Big Five Personality

The 44-item Big Five Inventory (BFI; John et al., 1991) was used to measure the five broad personality traits. All items were evaluated on a 5-point Likert scale, ranging from “strongly disagree” to “strongly agree.” Coefficient α reliabilities for the five trait scales in the present study were 0.707 for extraversion, 0.712 for agreeableness, 0.729 for conscientiousness, 0.706 for neuroticism, and 0.733 for openness. The Chinese version of BFI we used had been translated from English using common back-translation procedures (Brislin, 1970; Li and Chen, 2015), and the validity had been conformed in previous studies (Zhou, 2010; Li and Chen, 2015).

#### Social Support

Participants rated their social support from Chen and Yu (2019) using scales that ranged from 1 (strongly disagree) to 5 (strongly agree). The measure comprised three items, such as “It is easy for me to find someone to help when I meet with difficulties.” The entire survey demonstrated good reliability (α = 0.733).

#### Social Well-Being

Social well-being was measured through Keyes’s (1998) 15-item scale composed of five dimensions: social actualization, social integration, social acceptance, social contribution, and social coherence. Responses to this measure were assessed on a 5-point scale, from “strongly disagree” to “strongly agree.” An example of measure items was “I believe that people are kind.” The reliabilities of five dimensions were good (ranging from 0.702 to 0.725), and overall α reliability for the present sample was 0.791. Previous studies had confirmed the validity of social well-being measurement of Chinese version we used (Miao and Wang, 2009; Chen and Yu, 2019; Chen et al., 2020).

## Results

### The Common Method Bias Examination

As one of the main sources of measurement error, common method variance is a potential problem, which may be a threat to the validity of the conclusions. We tested for common method bias with a single-factor measurement model by combining all items into a single factor (Podsakoff et al., 2003; Rhee et al., 2017). Results showed a poor model fit [Comparative Fit Index (CFI) = 0.763, Tucker-Lewis Index (TLI) = 0.695, Goodness-of-Fit Index (GFI) = 0.719, Root Mean square Residual (RMR) = 0.025, Root Mean Square Error of Approximation (RMSEA) = 0.109]. The above results suggested that there was no common method bias effect.

### Descriptive Statistics and Correlations Between the Study Variables

There is no significant difference between the five different districts in Kunming. The correlation coefficients, means, and standard deviations are shown in Table 1. All the big five personality traits were correlated significantly with social support and five domains of social well-being (expect agreeableness and social coherence). Extraversion, agreeableness, conscientiousness, and openness were correlated positively with domains of social well-being (expect agreeableness and social coherence) and social support, whereas neuroticism correlated negatively with domains of social well-being and social support.

**TABLE 1:** Correlations, means, and standard deviations of all study variables.

	1	2	3	4	5	6	7	8	9	10	11
(1) Extraversion	1										
(2) Agreeableness	0.251***	1									
(3) Conscientiousness	0.265***	0.384***	1								
(4) Neuroticism	−0.478***	−0.334***	−0.361***	1							
(5) Openness	0.390***	0.232***	0.219***	−0.203***	1						
(6) Social integration	0.232***	0.247***	0.177***	−0.206***	0.191***	1					
(7) Social acceptance	0.169***	0.329***	0.176***	−0.220***	0.179***	0.330***	1				
(8) Social actualization	0.169***	0.261***	0.227***	−0.226***	0.131***	0.286***	0.423***	1			
(9) Social coherence	0.153***	0.042	0.137***	−0.253***	0.164***	0.178***	0.111***	0.249***	1		
(10) Social contribution	0.164***	0.241***	0.280***	−0.181***	0.240***	0.235***	0.269***	0.345***	0.177***	1	
(11) Social support	0.205***	0.120***	0.109***	−0.224***	0.209***	0.284***	0.189***	0.145***	0.206***	0.190***	1
M	3.220	3.697	3.354	2.836	3.265	3.288	3.483	3.463	2.836	3.727	3.470
SD	0.565	0.503	0.531	0.542	0.498	0.654	0.632	0.724	0.647	0.744	0.651

*^∗∗∗^*p* ≤ 0.001, ^∗∗^*p* ≤ 0.01, ^∗^*p* ≤ 0.05.*

### Regression Analyses

Statistical analyses were conducted with the Statistical Package for Social Sciences (SPSS, version 22.0). Based on preliminary analyses, multiple regression analyses were conducted to assess the relationship between the big five personality domains and dimensions of social well-being. Both gender and age were statistically controlled during the regression analysis, because there is evidence to show that social well-being likely increases with one’s age (Chen and Li, 2014) and that men generally score higher on well-being than women do (Miao and Wang, 2009). OLS regression was used to test the hypothesis. In each regression analysis, one social well-being dimension was entered as the dependent variable; gender, age, and all five personality domains were entered as potential predictors. Results of the regression analyses are presented in Table 2. Five personality traits were significant predictors of overall social well-being. Extraversion (β = 0.052, *p* ≤ 0.05), agreeableness (β = 0.197, *p* ≤ 0.001), conscientiousness (β = 0.138, *p* ≤ 0.001), and openness (β = 0.156, *p* ≤ 0.001) are positively related to social well-being, whereas neuroticism (β = −0.171, *p* ≤ 0.001) is negatively related to social well-being. H1_a_, H1_b_, H1_c_, H1_d_, and H1_e_ are supported. Extraversion (β = 0.118, *p* ≤ 0.001), agreeableness (β = 0.162, *p* ≤ 0.001), neuroticism (β = −0.065, *p* ≤ 0.05), and openness (β = 0.086, *p* ≤ 0.001) were significant predictors of social integration. Agreeableness (β = 0.268, *p* ≤ 0.001), neuroticism (β = −0.102, *p* ≤ 0.001), and openness (β = 0.089, *p* ≤ 0.001) were significantly associated with social acceptance. Agreeableness (β = 0.168, *p* ≤ 0.001), conscientiousness (β = 0.111, *p* ≤ 0.001), and neuroticism (β = −0.110, *p* ≤ 0.001) predicted social actualization significantly. Agreeableness (β = −0.088, *p* ≤ 0.001), conscientiousness (β = 0.060, *p* ≤ 0.05), neuroticism (β = −0.241, *p* ≤ 0.001), and openness (β = 0.125, *p* ≤ 0.001) were found to be predicting social coherence. Agreeableness (β = 0.120, *p* ≤ 0.001), conscientiousness (β = 0.191, *p* ≤ 0.001), and openness (β = 0.164, *p* ≤ 0.001) were found to be predictors of social contribution.

**TABLE 2:** Results of regression analyses for five personality traits predicting dimensions of social well-being.

	Social well-being	Social integration	Social acceptance	Social actualization	Social coherence	Social contribution
	**Model 1**	**Model 2**	**Model 3**	**Model 4**	**Model 5**	**Model 6**
Gender	0.005 (0.019)	0.034 (0.031)	0.030 (0.030)	0.032 (0.034)	−0.058* (0.031)	−0.020 (0.035)
Age	−0.025 (0.001)	−0.069** (0.001)	−0.021 (0.001)	0.021** (0.002)	0.027 (0.001)	−0.038* (0.002)
Extraversion	0.052* (0.020)	0.118*** (0.033)	0.016 (0.031)	0.034 (0.036)	−0.002 (0.033)	0.003 (0.037)
Agreeableness	0.197*** (0.021)	0.162*** (0.034)	0.268*** (0.033)	0.168*** (0.038)	−0.088*** (0.034)	0.120*** (0.039)
Conscientiousness	0.138*** (0.021)	0.045 (0.034)	0.015 (0.032)	0.111*** (0.037)	0.060* (0.034)	0.191*** (0.038)
Neuroticism	−0.171*** (0.021)	−0.065* (0.035)	−0.102*** (0.033)	−0.110*** (0.038)	−0.241*** (0.034)	−0.041 (0.039)
Openness	0.156*** (0.021)	0.086*** (0.034)	0.089*** (0.032)	0.034 (0.038)	0.125*** (0.034)	0.164*** (0.038)
*F*	69.353***	28.044***	36.090***	29.517***	24.751***	34.386***
*R*^2^	0.227	0.106	0.133	0.111	0.095	0.127
Δ*R*^2^	0.224	0.103	0.129	0.108	0.091	0.124

*Coefficients are standardized betas. ^∗∗∗^*p* ≤ 0.001, ^∗∗^*p* ≤ 0.01, ^∗^*p* ≤ 0.05.*

### Mediation Analyses

Further, mediation analysis was performed to determine whether the effect of big five personality on social well-being was mediated by social support. Mediation analyses were conducted following the recommendations of Preacher and Hayes (2004), using the PROCESS macro (version 3.0), developed by Hayes (2013). The current study used 5,000 bootstrapped samples with a 95% confidence interval. The results of this analysis are shown in Table 3. The results suggested five personality traits are related to social support significantly, and social support is positively related to social well-being. In addition, social support mediated the relationship between five personality traits and social well-being. H2_a_, H2_b_, H2_c_, H2_d_, and H2_e_ are supported.

**TABLE 3:** Summary of mediation analyses on five personality traits and social well-being (5,000 bootstraps).

Independent variable	Dependent variable	*c*	*c*’	*a*	*b*	*a* * *b*	Lower	Upper
Extraversion	Social well-being	0.215*** (0.018)	0.176*** (0.018)	0.224*** (0.028)	0.175*** (0.016)	0.039*** (0.006)	0.027	0.052
Agreeableness	Social well-being	0.306*** (0.020)	0.280*** (0.019)	0.141*** (0.032)	0.181*** (0.015)	0.026*** (0.006)	0.014	0.039
Conscientiousness	Social well-being	0.261*** (0.019)	0.242*** (0.018)	0.096** (0.030)	0.189*** (0.015)	0.018** (0.006)	0.008	0.029
Neuroticism	Social well-being	−0.272*** (0.019)	−0.232*** (0.018)	−0.238*** (0.029)	0.166*** (0.015)	−0.040*** (0.006)	−0.054	−0.029
Openness	Social well-being	0.238*** (0.021)	0.189*** (0.020)	0.287*** (0.031)	0.173*** (0.016)	0.050*** (0.007)	0.036	0.065

*c: Total effect; *c*’: direct effect; *a*: path from independent to mediator variable; *b*: path from mediator to dependent variable; *a*^∗^*b*: indirect effect; indirect effect; lower/upper: the 95% confidence interval of the indirect effect.^∗∗∗^*p* ≤ 0.001, ^∗∗^*p* ≤ 0.01, ^∗^*p* ≤ 0.05.*

## Discussion and Conclusion

The results obtained from the survey of 1,658 Chinese residents demonstrated the effects of five personality traits on five dimensions of social well-being and the mediating role of social support in the associations between big five personality and social well-being.

### Theoretical Contributions

Research on linkages between big five personality domains and five dimensions of social well-being conducted in China will likely contribute to the extant personality and well-being literature. First, this study provides empirical evidence about the relationship between big five personality and social well-being. The association between the big five personality and social well-being was evidenced in our study. However, our research also showed some inconsistencies with previous researches (Joshanloo et al., 2012). From our results, extraversion was significantly related to social integration; agreeableness was positively related to all five dimensions of social well-being; conscientiousness was positively related to social actualization, social coherence, and social contribution; neuroticism was negatively related to social integration, social acceptance, social actualization, and social coherence; openness was positively related to social integration, social acceptance, social coherence, and social contribution. This inconsistency may be explained by the fact that the differences between Iran and China. For instance, Iran is a non-Arab Muslim country; the interactions in Iran are regulated partly by religious norms (Joshanloo et al., 2012). In China, with the Reform and Opening, the way of thinking and behavior of Chinese are becoming more and more open and innovative (Ma, 2013). The goal of community construction in China is to establish the autonomous system of community residents (Fei, 2002). Community residents’ committee is an important organization of residents’ self-governing and self-service (Sun, 2016). Thus, most community residents can participate in community management and satisfy their own service needs via residents’ committee, which will benefit residents’ life quality.

Second, the study highlights the effect of social support on social well-being. The existing literature has shown the relationship between social support and subjective well-being or psychological well-being (Jasinskaja-Lahti et al., 2006; Brannan et al., 2013). Further, our study demonstrated social support is positively related to social well-being. Well-being is increasingly being associated with social and cultural relationships (Helliwell and Putnam, 2004). Community in China is increasingly becoming a place for residents to integrate into urban society (Chen et al., 2020). One of the most important responsibilities of the community is to achieve the society reconstruction (Fei, 2002). Thus, during the development of community, the Chinese government was committed to improving the quality of community services, which may provide more opportunities for residents to get more social support. Individuals having high social support means they had selected and built large and effective social networks, which can help to overcome difficulties in lives. With the help from their social relations, they will give a high appraisal to their circumstances and functioning in society; their social well-being also increases.

Third, the mediating effects were found for social support for relation between extraversion/agreeableness/conscientiousness/neuroticism/openness and social well-being. This may contribute to the literature on the relationship between big five personality and social well-being (Hill et al., 2012; Joshanloo et al., 2012). Previous studies neglected to examine the relationship and the mechanism between big five personality and social well-being from the perspective of the community. Community is an important place for residents’ daily activities. Individuals with different personality traits may build their social relations in different ways. Friends or family or neighbors around them may behave with different reactions. The different levels of social support will influence their evaluation of the social world, which may cause different levels of social well-being.

### Practical Implications

Our study provides valuable insight into how individuals of different traits to improve their social well-being. Social support serves as a mediator in the relationship between big five personality and social well-being. The results also affirm the importance of social support that can enhance social well-being. When one’s psychological, social, and/or resource needs are met, one is likely to experience greater social support, which is important for their well-being. Therefore, it is possible for residents to promote social support. Individuals should spend more time participating in community public affairs or other social activities that could offer opportunities for them to establish meaningful relationship with neighbors or friends.

### Limitations and Future Research

Despite these findings, our research is not without limitations. First, culture is an important factor that can influence both personality traits and well-being (Diener et al., 2003; Hofstede and McCrae, 2004). Our study just discussed the mediating effect of social support between personality and social well-being. Future research should explore the effects of different cultural variables (such as power distance, collectivism/individualism etc.,). In addition, comparative studies among different countries or regions are needed. Second, the cross-sectional design means that no causal conclusions for the found relationship can be made. Consequently, future researches should adopt longitudinal or experimental design to ascertain the relationship. Third, social support has usually been classified into several specific forms, such as informational support, emotional support, perceived social support (Taylor, 2011). In current study, we just regarded perceived social support as the mediating variable. So, future research should examine the effects of different forms of social support.

### Conclusion

The research used a sample drawn from 1,658 Chinese residents to investigate the relationship between big five personality and social well-being and the mediating effect of social support in the relationship between big five personality and social well-being. Results of this study support previous studies that highlighted the relationship between big five personality and social support (Swickert et al., 2010; Barańczuk, 2019). In addition, this study demonstrated the effects of five personality traits on five dimensions of social well-being. Lastly, the results demonstrated the mediating role of social support in the associations between extraversion/agreeableness/conscientiousness/neuroticism/open ness and social well-being, respectively.

## Data Availability Statement

The raw data supporting the conclusion of this article will be made available by the authors, without undue reservation.

## Ethics Statement

The studies involving human participants were reviewed and approved by Yunnan University of Finance and Economics. Written informed consent for participation was not required for this study in accordance with the national legislation and the institutional requirements.

## Author Contributions

YY, YZ, and JL designed the research and wrote the manuscript. YY and YZ are co-first authors of the article. All authors planned and conducted the data collection. YZ, JZ, and DL analyzed the data and revised the manuscript. All authors listed have made direct and intellectual contribution to the article and approved the final version for publication.

## Conflict of Interest

The authors declare that the research was conducted in the absence of any commercial or financial relationships that could be construed as a potential conflict of interest.
